# Has the Resignation of Independent Directors Holding Government Positions Improved Firm Performance?—A Quasi-Natural Experiment From China

**DOI:** 10.3389/fpsyg.2021.825366

**Published:** 2022-01-31

**Authors:** Tingting Zhang, Yanxi Li, Deshuai Hou

**Affiliations:** ^1^School of Business, East China University of Science and Technology, Shanghai, China; ^2^School of Economics and Management, Dalian University of Technology, Dalian, China; ^3^School of Accounting, Capital University of Economics and Business, Beijing, China

**Keywords:** independent director, firm performance, corporate governance, DID model, GID resignation

## Abstract

The Organization Department of the Communist Party of China (CPC) announced the *Opinions on Further Regulation on Party and Political Leaders and Cadres Working Part-Time (Holding Offices) in Enterprises* to force the resignation of government officials holding the position of independent director in listed companies (GID). This paper empirically examines the impact of the GID resignation on firm performance using a difference-in-differences (DID) model, which is an exogenous event with a “natural experiment.” The study finds that after the promulgation of the *Opinions*, firms that lose some of their political resources and their corporate performance decreases significantly compared to firms that do not experience GID resignations. A good external governance environment, while somewhat weakening, is not sufficient to offset the negative impact of the loss of political resources on firm performance. This paper further explores the mechanism by which the GID resignation affects firm performance: one important way in which the resignation of GIDs cause the loss of political resources on which the firm's development depends is that the loss of the firm's tax benefits after GID resignation directly leads to a decline in performance; it also leads to a reduction in the firm's financial subsidy income and a reduction in the amount of bank loans, but both of these do not have a significant effect on the decline in firm performance. The study suggests that GIDs play more of a resource-providing “official” role than an “independent director's” supervisory and advisory role in Chinese listed companies. The findings of this paper reveal the phenomenon of “Political-Business Spin” in China, which has some implications for developing countries, represented by China, to improve the independence of the board of directors and the corporate governance.

## Introduction

The Organization Department of the Central Committee of the Communist Party of China (CPC) issued *Document No. 18: Opinions on Further Regulation on Party and Political Leaders and Cadres Working Part-Time (Holding Offices) in Enterprises* (hereafter referred to as the *Opinions*) on October 19, 2013. The *Opinions* highlight that the highly controversial group of government officials holding the position of independent director in listed companies (GID) was gradually withdrawing from China's capital market. An intensive wave of departures of GIDs from Chinese listed companies occurred immediately following the release of the *Opinions*. As of the end of December 2016, which was more than 3 years after the promulgation of the *Opinions*, a total of 2,255 independent director (ID) resignations were issued in China's capital market. Among these announcements, 1,046 directly or indirectly mentioned in the announcements due to the *Opinions*^1^, highlighting the impact that the *Opinions* had on ID appointment in China's listed companies. In this paper, we focus on the impact of the mandatory resignation of GIDs, which is an exogenous event with considerable “natural experiment” value, on long-term firm performance and extensively analyzed the intrinsic mechanisms that influence firm performance.

The China Securities Regulatory Commission promulgated the *Guidelines for the Introduction of Independent Directors into Listed Companies* on August 16, 2001, formally launching the ID system into China's capital market. Adams et al. (2010) asserted that the supervisory and advisory functions of IDs enhance corporate value and that these functions are guaranteed by the professionalism and independence of IDs (Fama and Jensen, 1983). In China's capital market, major shareholders often engage in tunneling to encroach on the interest of medium and small shareholders (Jiang et al., 2010).

*Company Law of the People's Republic of China* regulations state that IDs are responsible for protecting the legal interests of minority shareholders. In reality, the appointment of government officials to serve as IDs in publicly trading companies typically bears political overtones (Fracassi and Tate, 2012). In a capital market that follows the “all is permissible unless prohibited by law” ideology, appointing IDs with political backgrounds seems to be a strategy that companies employ to establish political ties and acquire government resources for corporate development. Moreover, GIDs are not exclusive to China. The “revolving door” phenomenon also exists in developed Western countries, where retired officials accept positions in firms or institutions after retirement. Under a robust legal framework, government–industry cooperation can facilitate the creation of wealth. However, “dummy” or unspecialized IDs are often appointed in China's capital market, leading to the common belief that the appointment of GIDs is a form of “political contribution.” Investors' doubts concerning the appointment of retired government officials for ID positions in firms warn that the accumulative influence of government officials facilitates rent seeking, consequently interfering with the fair trade mechanics of the market. The release of the *Opinions* targeted dual-status IDs and forced GIDs to resign. This study elucidates the effects that this wave of GID resignation had on firm performance, to determine whether the appointment of GIDs improved corporate governance.

**Figure 1:**
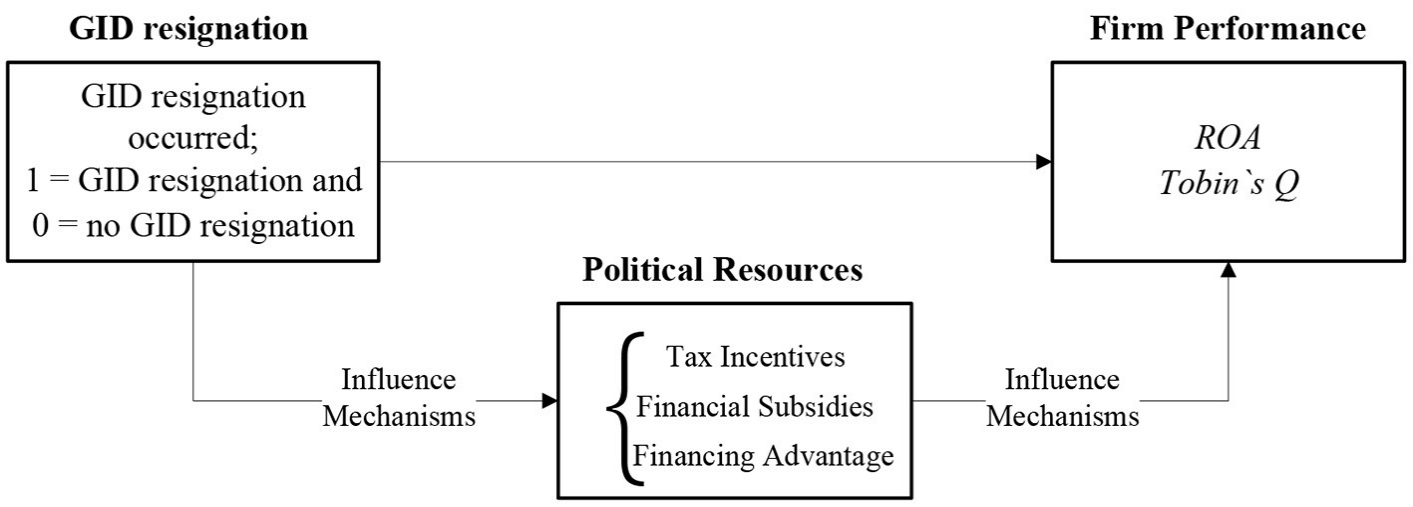
Research logic diagram.

The main contributions of this paper are: first, based on the exogenous event of the promulgation of the Opinions as a “quasi-natural experiment,” this paper uses panel data for 14 periods before and after the promulgation of the Opinions. We use a DID model to empirically test the impact of the GID resignation on firm performance and examine the real implementation effect after the promulgation of the Opinions. In contrast, a number of the existing studies in China use event study to examine the market reaction to GID resignation, which is essentially an investor's expectation (perception) of whether GIDs can play a role in the emerging market, not the real implementation effect after the promulgation of the Opinions. Second, this paper takes the promulgation of the Opinions as an opportunity to adopt a DID model, which not only can more accurately measure the impact of the GID resignation on corporate performance, but also can effectively weaken the possible endogeneity problems in the study and ensure the reliability of the research findings. On the one hand, when previous literature studying independent directors encountered the endogeneity problem, only a small amount of literature used instrumental variables to mitigate the effect of endogeneity, but most of the literature did not take any measures. Although using instrumental variables to mitigate the endogeneity problem is a proven method, the validity of instrumental variables needs to strictly satisfy both “relevance” and “exogeneity” conditions. It is very difficult to find a valid and appropriate instrument in the field of financial accounting. On the other hand, the problem of endogeneity in the area of independent directors' resignation is mainly due to the possible self-selection of the sample arising from the “active” resignation of the independent directors, and it is difficult to distinguish the reasons for the resignation of the independent directors in the existing literature. Third, this paper finds that the main reason for the decline in corporate performance due to the resignation of GIDs is the loss of political resources on which corporate development depends. This study uncovers the mechanisms and paths by which GIDs affect corporate performance, provides direct empirical evidence that Chinese listed companies rely on GIDs to obtain political resources. Figure 1 illustrates the research logic.

The remainder of this paper is structured as follows. Chapter 2 reviews the literature on the effects of board independence on firm value and corporate governance, providing a theoretical basis for analysis, and hypothesis testing. Chapter 3 presents the research design, including DID model settings, sample selection, data sources, and variable definitions. Chapter 4 presents the empirical testing and analysis. The final chapter presents the research conclusion and implications.

## Theoretical Analysis and Hypothesis Formulation

Existing studies have largely focused on the effects of board independence on firm value and corporate governance. Although foreign scholars have already extensively discussed this topic, severe endogeneity problems exist in most of these studies. The relationship between board independence and firm value is determined endogenously, as though the independence or structure of the board affects firm value; however, firm value might affect board independence or structure. Foreign scholars have adopted certain exogenous policies to explain these problems. For example, Goldman et al. (2009) asserted that companies with IDs that possess strong political affiliations typically have higher stock returns than those without.

The article also analyses the 2000 US presidential election and found that companies with IDs connected to the Republican Party increased in value, whereas companies connected to the Democratic Party decreased in value. Duchin et al. (2010) examined a series of policies focused on enhancing board independence to determine the relationship between board independence and firm performance. The researchers found that when the cost of acquiring information was low, an increase in board independence facilitated firm performance. Controlling for a series of endogenous problems, Liu et al. (2015) found a significant and positive correlation between board independence and firm performance. Francis et al. (2015) found a significant and positive correlation between academic directors and company earnings. Beasley (1996) found that large proportions of outside members on the board of directors significantly reduced financial statement fraud. Dewally and Peck (2010) examined the premature resignation behavior of IDs, asserting that such behavior signifies that the company's corporate governance is flawed and that IDs engage in such behavior to evade risk and for self-preservation. Currently, exogenous variables that reflect the real-world conditions in China are lacking, making endogeneity problems unavoidable in relevant research. However, the promulgation of the *Opinions* on October 19, 2013 by the Organization Department of the CPC, which caused GIDs to resign, provided an opportunity to conduct a natural experiment, enabling us to accurately measure the effects of GID resignation on firm performance and resolve existing critical endogeneity problems.

After the Organization Department of the CPC issued Document 18 on October 19, 2013, Chinese scholars centered their research on the effects of GID resignation on stock market responses. Most of the scholars that had previously used an event study approach to examine the effects of GID focused on investors' predictions concerning whether GIDs would affect emerging markets rather than on the realistic effects of the promulgation of the *Opinions*. Existing studies have neither explored the effects of GID resignation after the promulgation of the *Opinions* on long-term firm performance, nor extensively analyzed the intrinsic mechanisms that influence firm performance.

Resource dependence theory states that the heterogeneity of firm resources is the core factor influencing competitive advantage and performance. However, firms typically do not control the critical, yet scarce, resources required for survival and development. Therefore, they take measures to strengthen their control of crucial external resources and reduce uncertainty and risk. Eastern societies are largely relationship-based, and political affiliations are considered a key resource for firm development. The competitive advantage generated through political affiliations positively affects firm value and incites the “resource effect.” Numerous studies have mentioned that political affiliations alleviate the financing constraints of firms (Khwaja and Mian, 2005), making it easier to secure government subsidies (Faccio, 2006), and tax incentives (Claessens et al., 2008) and enhancing firm performance and stock value. In transition economies, government officials have greater power and flexibility to curb policies, allocate resources, and regulate industries. Therefore, firms that seek to transcend industry barriers and acquire fair trade opportunities are required to establish positive political ties with the government. Appointing government officials as IDs of a firm is a legal and convenient means for entrepreneurs to overcome the limitations of their personal social networks and relationships while simultaneously creating opportunities to lobby GIDs for power.

The creation of the ID system was initially to ensure the integrity of corporate governance structures. However, this Western ideology has become shrouded with government–business collusion in China, where IDs with a background in politics mediate between the business and the government. GIDs exploit their current or former “official” status and draw on the relationships and resources established during their time in office to build favorable political ties between their firm and the government. GIDs not only possess a keen eye for politics and rich political and human resources but also hold higher social status and conversation dominance compared with other professional IDs (e.g., accounting, legal, or analysis professionals), who possess a keen eye for investment and vast knowledge and skill. The professionalism and independence of IDs ensures their supervisory and advisory functions in the firm. By comparison, the value of GIDs stems from their political background, which facilitates resource acquisition and rent seeking, rather than benefiting supervisory and advisory functions. China is the world's largest transition economy, and rent seeking is common in emerging markets. Firms engage in non-productive competition to gain a monopoly advantage and scarce resources provided by the company, thereby enhancing their competitive advantage and supernormal profits. The political background and underlying political resources of GIDs facilitate their firms in enhancing rent-seeking benefits and reducing rent-seeking costs, creating an advantage unmatched by professional IDs. To some extent, this “government favoritism” effect is an effective alternative mechanism for protecting weak investors, positively influencing firm performance. Therefore, firms with GIDs in their board of directors are able to gain a stronger competitive advantage and exhibit better firm performance than those without.

However, after the promulgation of the *Opinions* by the Organization Department of the CPC, government officials were no longer eligible to serve as IDs. GIDs voluntarily withdrew from their positions on firm boards because they valued their political careers and for self-preservation. Withdrawal from firm payrolls meant that GIDs no longer had incentives to establish political ties or acquire scarce resources on behalf of the firm. When GIDs successively resigned after the promulgation of the *Opinions*, firms were no longer able to gain these competitive advantages. The promulgation of the *Opinions* caused firms to lose a portion of their political affiliations and resources tethered to the GIDs, with consequent negative impacts on firm performance. Therefore, we proposed the following hypothesis:

- **Firms with GID resignation experienced a steeper drop in firm performance than those without GID resignation after the promulgation of the *Opinions***.

## Research Design

### DID Model Settings

Ashenfelter (1978) introduced the DID model, which is commonly applied in the natural sciences, to Western economics. Thereafter, the model became a widely accepted instrument for scholars for evaluating policy effectiveness. In the model, the promulgation of a specific policy at a specific time is viewed as an external stimulus. To observe the effects of the preceding stimulus, the sample is divided into a treatment group, comprising entities affected by the policy, and a control group comprising entities unaffected by the policy. The changes in the treatment and control groups before and after the promulgation of the policy are observed to accurately evaluate the effects of the policy. Therefore, when analyzing panel data, DID models can control the effects of unobservable heterogeneous factors that exist between sample firms and the unobservable general factors that change over time, thereby generating unbiased results (Abadie, 2005). Conversely, using ordinary-least-square models would significantly overestimate policy effects. The forced GID resignation event that occurred following the promulgation of the *Opinions* would cause discrepancies in the firm performance data before and after GID resignation, as well as discrepancies in the firm performance data between companies with and without GID resignation in the same period.

The DID model adopted in this study created excellent natural experiment conditions for evaluating the effects that GID resignation had on firm performance. Notably, a vital precondition of using the DID model to evaluate policy changes is that the policy itself must be exogenous and unrelated to residuals of the model. From a regulatory perspective, the intention of the *Opinions* was to prevent the problem of rent seeking driven by party and government officials. Therefore, GID resignation, which resulted in the loss of political affiliations established by GIDs, is a completely exogenous event. The *Opinions* deprived firms of their power over their GIDs. Therefore, to prevent the occurrence of endogenous problems, a random sampling method was adopted in this study to select the treatment and control groups.

A DID model was designed based on the research objectives, where firm performance represents firm performance, *T* = 1 represents before GID resignation and *T* = 0 represents after, *D*_*i*_ = 1 represents the *i*^th^ firm affected by the policy stimulus with GID resignation and *D*_*i*_ = 0 represents the same without GID resignation, and Treated represents whether GID resignation occurred in a specific firm at a specific period because of the promulgation of the *Opinions*. Based on this design, we know that GID resignation cannot occur in the 0th period and that GID resignation only occurred in the *i*^th^ firm (*D*_*i*_ = 1) in the 1st period. Therefore, Treated = $D_{i}^{*}$T. Using these inferences, the DID Model (1) can be expressed as follows:


(1) $$\begin{array}{l} Firm\;Performance_{it}=\alpha D_{i}+\beta T+\gamma \left(D_{i}*T\right)+u_{it} \end{array}$$

**Figure 2:**
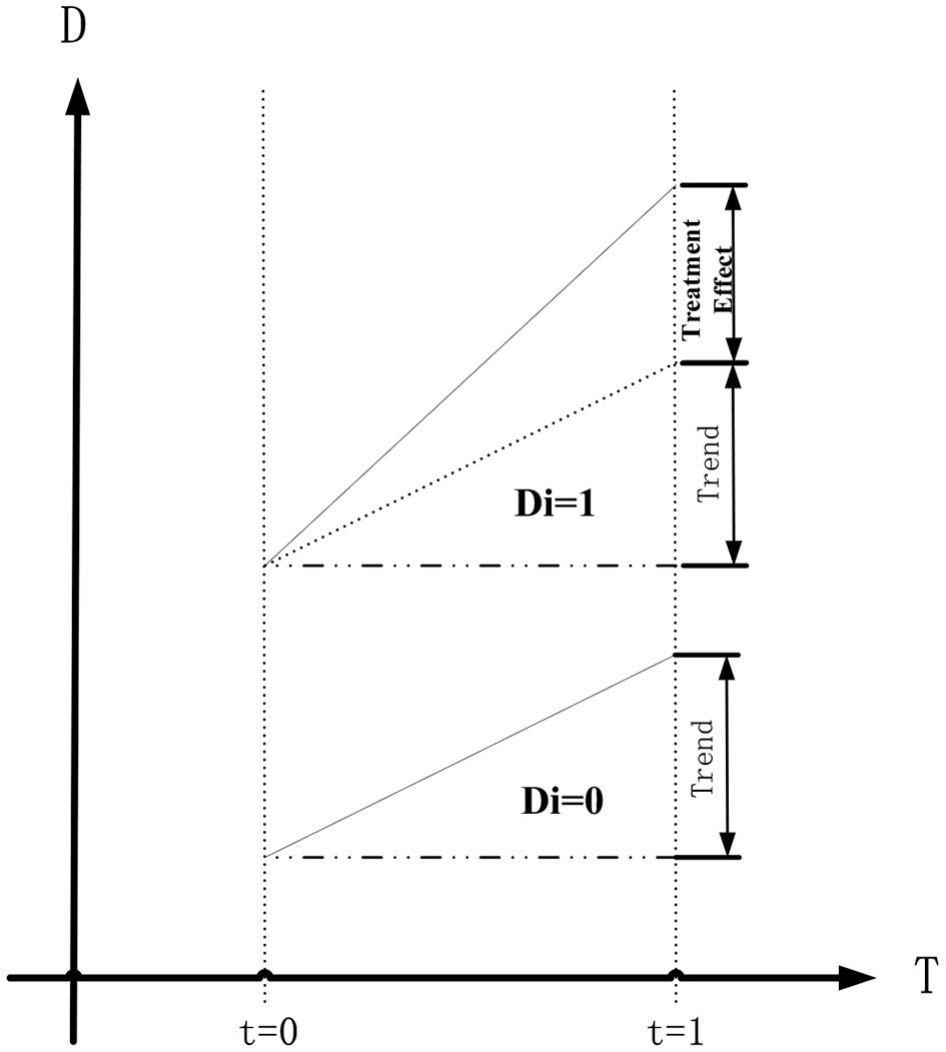
The treatment effect for evaluation of DID model.

Calculating the time difference of Model 1 yielded the following model:


(2) $$\begin{array}{l} \Delta Firm\;Performance_{t}=\beta +\gamma D_{i}+\Delta u_{it} \end{array}$$

and the following was obtained by calculating the time difference of Model 2:


(3) $$\begin{array}{l} E\left(\Delta Firm\;Performance_{1}-\Delta Firm\;Performance_{0}\right)=\gamma \end{array}$$

where γ represents the estimated treatment effect. Therefore, the interaction term coefficient (γ) obtained from the regression of Model (1) is the treatment effect for evaluation in this study (Figure 2).

Although the policy stimulus forcing GID resignation is exogenous, firm performance is also affected by other factors. These factors could cause a pretrend effect on the firm performance of the treatment and control groups, consequently influencing the effectiveness of DID estimations. Therefore, we incorporated a number of control variables (the definitions of the variables are stated in **Table 2**) that were likely to affect firm performance in the base DID model and controlled the industry and season effects. The following model was created:


(4) $$\begin{array}{l} Firm\;Performance_{it}=\alpha D_{i}+\beta T_{i}+\gamma (D_{i}*T_{i})\\ \;+\delta Control\;Varibles_{it}+u_{it} \end{array}$$

In Model (4), the explained variable *Firm Performance*_*it*_ represents the firm performance of the *i*^th^ firm in the *t*^th^ period. This variable can be measured using the indices *ROA* and *TobinQ*. *D*_*i*_ represents whether GID resignation occurred in the *i*^th^ firm and *T*_*i*_ represents whether the period was before or after the promulgation of the *Opinions*; *T*_*i*_ = 1 represents that the sample firm was observed after Q4 of 2013; otherwise, *T*_*i*_ = 0. *Control Varibles* represents all the variables that could potentially affect the model, including firm size, financial leverage, firm growth, duality, board size, board independence, and ID externality, whereas *u*_*it*_ represents model disturbance.

### Sample Selection and Data Sources

All explained variable and control variable data were acquired from the China Stock Market and Accounting Research (CSMAR) Database. GID resignation data were acquired from the “Firm Announcements” column in the Wind Database. We manually searched for ID resignation announcements released by A-share for non-financial firms in China after October 19, 2013, finding 2,255 announcements^2^. A review of these announcements revealed that 1,046 directly attributed resignation to the *Opinions*. A total of 1,709 valid GID resignation announcements remained after the screening process. Further observations showed that the 1,709 announcements were released by 1,281 firms, implying that after the promulgation of the *Opinions* (between October 19, 2013 and December 31, 2016), GID resignation occurred in 1,281 listed companies in China. The screening results are stated in Table 1.

**Table 1:** Screening results of ID resignation announcements from 2014 to 2016.

**Sample screening process**	**Exclusions**	**Announcements**
Panel A: Statistics on ID Resignation Announcements from 2014 to 2016		
Non-finance firms:		2,255
– Special treatment (ST; ^*^ST)	−57	2,198
– Term expiration^a^	−141	2,057
– Personal reasons (including age and health)	−214	1,843
– Work reasons^b^	−104	1,739
– Other reasons (including no reason given)	−30	1,709
GID resignation announcements		1,709
Announcements that directly attributed resignation to the *Opinions*		1,046
Panel B: Statistics on GID Resignations from 2014 to 2016		
GID resignation announcements		1,709
- Firms that released multiple resignation announcements	−428	1,281
Firms that released GID resignation announcements after the promulgation of the *Opinions*		1,281

a *According to the regulations stipulated in the Company Law of the People, Company Policy, and Guidelines for the Introduction of Independent Directors into Listed Companies, the term of IDs shall be no longer than 6 years. We excluded the IDs who withdrew from their firms after their term expired to ensure the integrity of the research design*.

b *A considerable portion of the announcements did not clearly state the reason for resignation, simply stating “work reasons” or “personal reasons”. For these announcements, we manually searched the Wind Database, CSMAR Database, official websites of publicly trading companies, and Baidu to obtain the personal profiles of the IDs. If the ID possessed a background in politics and resigned after October 19, 2013, we assumed that the reason for resignation was the promulgation of the Opinions*.

* *ST refers to stocks listed in China with three consecutive years of operating losses and are subject to a delisting risk warning*.

The *Opinions* were announced by the Organization Department of the CPC on October 19, 2013. To compare the performance of firms with and without GID resignations in the same period and the changes in firm performance before and after the promulgation of the *Opinions*, we selected an observation period between 2011 and 2016. The treatment group comprised the publicly trading companies that released GID resignation announcements after the promulgation of the *Opinions*, whereas the control group comprised the publicly trading companies that did not. The experiment period was between 2014 and 2016 and the control period was between 2011 and 2013. We performed an initial screening of the sample according to good research practice guidelines. Specifically, we eliminated (1) finance and insurance firms, (2) ST and ^*^ST firms, and (3) firms with inadequate data. After the initial screening, 12,200 sample observations were obtained across the 6 years before and after the promulgation of the *Opinions*.

### Variable Definitions

#### Explained Variables

In previous studies on the finance and accounting domains, firm performance was determined based on accounting or market performance. Bowen et al. (2008) has asserted that the total return on assets (ROA) is a key indicator of asset performance. Therefore, total ROA was adopted in this study to measure the accounting performance of the sample firms, presented as the ratio of net profit to total assets. Francis et al. (2015) asserted that Tobin's Q is a market-based indicator for measuring the performance of publicly trading companies, presented as the ratio of market value to replacement cost of firm assets. Therefore, Tobin's Q was adopted in this study to measure the market performance of each sample firm.

#### Explanatory Variable

The purpose of this study was to determine whether the GID resignations caused by the promulgation of the *Opinions* influenced firm performance. Therefore, we set the promulgation of the *Opinions* as a break-off point for GID resignation. For each 3-month unit (Q), firms that released GID resignation announcements after October 19, 2013 were allocated a D-value of 1. All other firms were allocated with a *D*-value of 0.

#### Control Variables

Although the policy stimulus causing forced GID resignation is exogenous, firm performance can also be affected by other factors, which may influence the effectiveness of DID estimations. Based on common practice in empirical studies, we controlled the main factors that influence firm performance to minimize the biases caused by missing variables. We adopted the variables proposed by Duchin et al. (2010), namely firm size (*Lnsize*), financial leverage (*Lev*), firm growth (*Growth*), duality (*Dual*), board size (*Lnbrdsize*), board independence (*Indrratio*), and ID externality (*Indplace*), as the control variables in the model. We also controlled industry and season effects.

The definitions of variables are presented in Table 2.

**Table 2:** Variable definitions.

**Type**	**Symbol**	**Definitions and meanings**
Independent variables	*ROA*	Accounting performance: measured using total ROA and presented as a ratio between net profit and total asset
	*TobinQ*	Market performance: measured using Tobin's *Q* and presented as the ratio of the market value to the replacement cost of firm assets
Dependent variables	*D*	GID resignation (dummy variable): measures whether GID resignation occurred; 1 = GID resignation and 0 = no GID resignation
	*T*	Time period: 1 = a 3-month period after the promulgation of the *Opinions*, and 0 = periods before promulgation
Control variables	*Lnsize*	Firm size: presented as the natural logarithm of the total assets at the end of each quarter
	*Lev*	Financial leverage: measured using quarterly assets and liabilities and presented as the ratio of the total liabilities to the total assets
	*Growth*	Firm growth: measured using the sales revenue growth rate
	*Dual*	Duality: measures whether directors also serve as general managers; 1 = yes and 0 = no
	*Soe*	State-owned enterprises: 1 = state-owned firm; 0 = privately owned firm
	*Lnbrdsize*	Board size: measured using the natural logarithm of number of members on the board of directors
	*Indratio*	Board independence: measured using the number of ID on the board of directors.
	*Indplace*	ID externality (dummy variable): 1 = IDs working inside the publicly trading company; 0 = IDs working outside
	*Ind*	Industry (dummy variable): measured based on the industry classifications defined by the Chinese Securities Regulatory Commission in 2001; 20 industry dummy variables were selected
	*Year*	*Year* (dummy variable): presented as 6 dummy variables between 2011 and 2016

## Empirical Testing and Analysis

### Descriptive Statistics

The descriptive statistics of the variables used in this study are presented in Table 3, indicating the performance of the sample firms. The mean value for *ROA* (accounting performance) was 3.3%, and 2.176 for *TobinQ* (market performance). The mean value for *D* (GID resignation) was 0.371, suggesting that GID resignation occurred in 37.1% of the sample firms after the promulgation of the *Opinions*. These statistics explain that most of the publicly trading companies in China have or had employed government officials currently in office as an ID. They also explain that GIDs successively resigned due to regulatory pressure after the promulgation of the *Opinions*. The mean value for *Lev* (financial leverage) was 0.412, suggesting that the average debt-to-asset ratio in the sample firms was 41.2%. The mean value for *Growth* (sales revenue growth) was 0.137, suggesting that the sample firms achieved positive firm growth. The mean value for *Dual* (duality) was 0.259, suggesting that directors served as general managers in 25.9% of the sample firms and validating that duality is common practice in publicly trading companies in China. The mean value for *Soe* (state-owned enterprises) was 0.388, suggesting that 38.8% of the sample firms were state-owned enterprises. The mean, maximum, minimum, and median values for *Indratio* (ID proportion) were 0.373, 0.571, 0.333, and 0.333, respectively. These values suggest that on average, IDs occupied 37.3% of the board in publicly trading companies in Taiwan, which is higher than the requirement of 33% or higher. Nonetheless, most of the sample firms chose to maintain ID proportion at a statutory level. The mean value for *Indplace* (externality) was 0.526, suggesting that 47.4% of the IDs worked outside the sample firms and that publicly trading companies favored the employment for off-site IDs. To eliminate the effects of outliers on the robustness of the research findings, we Winsorized the continuous variables in the model with extreme quantiles (<1 or >99%).

**Table 3:** Descriptive statistics.

**Variables**	**Mean**	**Median**	**Max**	**Min**	**Sd**	***N***
*ROA*	0.033	0.023	0.172	−0.094	0.036	13,200
*TobinQ*	2.176	1.613	13.430	0.134	2.036	13,200
*T*	0.502	1	1	0	0.500	13,200
*D*	0.371	0	1	0	0.483	13,200
*Lnsize*	22.220	21.970	26.920	18.920	1.360	13,200
*Lev*	0.412	0.402	1.248	0.041	0.217	13,200
*Growth*	0.137	0.033	4.690	−0.840	0.636	13,200
*Dual*	0.259	0	1	0	0.438	13,200
*Soe*	0.388	0	1	0	0.487	13,200
*Lnbrdsize*	2.163	2.197	2.708	1.609	0.197	13,200
*Indratio*	0.373	0.333	0.571	0.333	0.054	13,200
*Indplace*	0.526	1	1	0	0.499	13,200

IDs were forced to resign after the promulgation of the *Opinions* by the Organization Department of the CPC on October 19, 2013. The descriptive statistics of the variables concerning the firms with and without GID resignations and their variance outcomes are presented in Table 4. Policy-enforced GID resignation did not occur until October 19, 2013; therefore, the indices of the treatment and control groups between 2014 and 2016 were compared. The results of this comparative analysis revealed that *TobinQ* for the treatment group was significantly lower than that of the control group, suggesting that, to some extent, the firms with policy-enforced GID resignation had a lower market value than those without. The *Lnsize* of the treatment group was 22.45, significantly higher than that of the control group (22.23), suggesting that larger firms were more likely to employ GIDs. The *Soe* of the treatment group was 0.419, significantly higher than that of the control group (0.345). Because the *Growth, Dual, Lnbrdsize*, and *Indplace* of the two groups were significantly different, these variables were controlled in the model design.

**Table 4:** Comparative analysis of statistics from 2014 to 2016.

	**Treatment group**				**Control group**				**Mean**	**Median**
	**Mean**	**Median**	**Sd**	***N***	**Mean**	**Median**	**Sd**	***N***	**t-stat**	**z-stat**
*ROA*	0.033	0.023	0.037	3,420	0.032	0.022	0.038	5,779	0.745	1.386
*TobinQ*	2.540	1.830	2.370	3,420	2.648	1.968	2.423	5,779	−2.083^**^	−2.937^***^
*Lnsize*	22.450	22.210	1.402	3,420	22.230	21.990	1.318	5,779	7.457^***^	7.458^***^
*Lev*	0.419	0.406	0.210	3,420	0.419	0.408	0.213	5,779	0.118	0.345
*Growth*	0.141	0.039	0.605	3,420	0.176	0.043	0.709	5,779	−2.426^**^	−0.855
*Dual*	0.236	0	0.425	3,420	0.276	0	0.447	5,779	−4.264^***^	−4.260^***^
*Soe*	0.419	0	0.494	3,420	0.345	0	0.476	5,779	7.103^***^	7.084^***^
*Lnbrdsize*	2.170	2.197	0.207	3,420	2.136	2.197	0.194	5,779	7.907^***^	6.822^***^
*Indratio*	0.374	0.357	0.055	3,420	0.374	0.333	0.053	5,779	0.085	−0.118
*Indplace*	0.541	1	0.498	3,420	0.523	1	0.500	5,779	1.655^*^	1.682^*^

***, **, * *denote coefficients significance at 1, 5, and 10%, respectively*.

### Correlation Testing

Pearson and Spearman correlation tests were performed on the main variables; the resulting correlation matrix is presented in Table 5. The upper triangular section of the matrix contains the Spearman correlation coefficients, and the lower triangular section contains the Pearson correlation coefficients. The table indicates that the correlation coefficients between variables were all below 0.6, suggesting that severe multiple collinearities were not present among the variables.

**Table 5:** Correlation matrix of the main variables.

	***ROA***	***TobinQ***	***Lnsize***	***Lev***	***Growth***	***Dual***	***Soe***	***Lnbrdsize***	***Indratio***	***Indplace***
*ROA*	1	0.342^***^	−0.152^***^	−0.362^***^	0.226^***^	0.058^***^	−0.156^***^	−0.031^***^	−0.020^***^	0.0167**
*TobinQ*	0.278^***^	1	−0.367^***^	−0.372^***^	0.039^***^	0.194^***^	−0.406^***^	−0.229^***^	0.035^***^	0.068^***^
*Lnsize*	−0.131^***^	−0.495^***^	1	0.462^***^	−0.025^***^	−0.243^***^	0.493^***^	0.288^***^	0.007	−0.074^***^
*Lev*	−0.346^***^	−0.395^***^	0.437^***^	1	−0.007	−0.174^***^	0.371^***^	0.181^***^	−0.009	−0.064^***^
*Growth*	0.104^***^	0.032^***^	−0.002	0.040^***^	1	0.023^***^	−0.038^***^	−0.015**	0.002	0.015**
*Dual*	0.048^***^	0.152^***^	−0.229^***^	−0.173^***^	0.012	1	−0.284^***^	−0.209^***^	0.104^***^	0.026^***^
*Soe*	−0.129^***^	−0.305^***^	0.495^***^	0.369^***^	−0.019^***^	−0.284^***^	1	0.294^***^	−0.040^***^	0.012
*Lnbrdsize*	−0.039^***^	−0.204^***^	0.297^***^	0.182^***^	−0.020^***^	−0.199^***^	0.306^***^	1	−0.468^***^	−0.032^***^
*Indratio*	−0.005	0.054^***^	0.041^***^	−0.005	0.004	0.116^***^	−0.040^***^	−0.476^***^	1	0.012
*Indplace*	−0.002	0.062^***^	−0.056^***^	−0.061^***^	0.019^***^	0.026^***^	0.012	−0.029^***^	0.013*	1

***, **, * *denote coefficients significance at 1, 5, and 10%, respectively*.

### Regression Results and Analysis

The outcomes of a regression analysis on the relationship between GID resignation and GP are presented in Table 6. Columns (1) and (3) present the regression results without the control variables, whereas firm characteristics, board characteristics, year, and industry were controlled in Columns (2) and (4). When the explained variable was ROA, D^*^T achieved a significant and negative correlation (10% level) with *ROA* without the control variables in Column (1) and a significant and negative correlation (1% level) with *ROA* (−0.003) with the control variables in Column (2). When the explained variable was TobinQ, D^*^T achieved a significant and negative correlation (5% level) with *TobinQ* without the control variables in Column (3) and a significant and negative correlation (1% level) with *TobinQ* (−0.173) with the control variables in Column (4). These results suggest that firms with GID resignation experienced a steeper drop in firm performance than those without GID resignation after the promulgation of the *Opinions*, supporting our hypothesis. Furthermore, the control variables had a strong explanatory power over the explained variables. *Lev* achieved a significant and negative correlation with the explained variables, suggesting that firm performance decreased concurrently with an increase in the debt-to-asset ratio. In addition, accounting performance increased and market performance decreased concurrently with an increase in firm size and ID proportion. Firms with directors serving as general managers had better market performance than those without, and state-owned enterprises had poorer firm performance than privately owned firms.

**Table 6:** Regression analysis for GID resignation and firm performance.

	**(1)**	**(2)**	**(3)**	**(4)**
	***ROA***	***ROA***	***TobinQ***	***TobinQ***
*T*	0.000	0.013^***^	0.916^***^	2.405^***^
	(0.51)	(10.13)	(24.71)	(40.64)
*D*	0.002^**^	0.003^***^	0.018	0.207^***^
	(2.43)	(3.77)	(0.41)	(6.42)
D*T	−0.002^*^	−0.003^***^	−0.126^**^	−0.173^***^
	(−1.70)	(−2.79)	(−2.06)	(−3.84)
*Lnsize*		0.005^***^		−0.462^***^
		(20.24)		(−38.25)
*Lev*		−0.076^***^		−2.342^***^
		(−49.78)		(−33.34)
*Growth*		0.000		0.000
		(0.88)		(1.34)
*Dual*		−0.001		0.063^**^
		(−1.06)		(2.38)
*Soe*		−0.004^***^		−0.137^***^
		(−6.77)		(−4.94)
*Lnbrdsize*		−0.004^**^		0.022
		(−2.56)		(0.31)
*Indratio*		−0.014^***^		1.781^***^
		(−2.68)		(7.49)
*Indplace*		−0.002^***^		0.001
		(−4.50)		(0.04)
*Ind*		Control		Control
*Year*		Control		Control
*_cons*	0.032^***^	−0.075^***^	1.733^***^	11.813^***^
*R^2^*	0.000	0.351	0.046	0.480
*Adj-R^2^*	0.000	0.349	0.046	0.479
*F*	2.092^***^	229.353^***^	293.436^***^	392.844^***^
*N*	13,200	13,200	13,200	13,200

*R^2^ indicates Goodness of Fit, Adj-R^2^ means adjusted R^2^, F represents an F-test value, N represents the number of regression samples*.

***, **, and * *denote coefficients significant at the 1, 5, and 10% levels, respectively (two-tailed test)*.

### Further Discussion

This section elaborates on the influence mechanism of GID resignation on firm performance. The presence of GIDs implies that firms have the capability to acquire specific political resources, which is greatly beneficial for reducing business risk. In emerging markets dominated by state-owned banks, GIDs can exploit their “government” status to secure bank loans for their firms, which serves as an implicit guarantee that reinforces investor confidence. Subsequently, banks are more inclined to provide credit to these firms. Khwaja and Mian (2005) found that political affiliations helped firms secure financial convenience. China's decentralization reform provided government officials with the freedom and power to decide whether to grant financial subsidies and tax incentives to firms. This created a platform for the rent seeking of GID power. Government officials hold considerable power and flexibility in deciding the provision of government subsidies and tax incentives, and GIDs can exploit the social networks they established using their current or previous government status to help their firm establish positive political ties and overcome the soft constraints of gaining government subsidies and tax incentives. Faccio (2006) found that firms with political affiliations are more likely to gain government funding. The “government” status of GIDs enables firms to expand their financial options and secure government subsidies and tax incentives at minimal cost. The forced resignation of GIDs following the promulgation of the *Opinions* meant that firms lost a portion of their political affiliations, implying the corresponding loss of a portion of the political resources they would have otherwise gained with the help of GIDs. Therefore, we selected tax incentives, financial subsidies, and the financing advantage as the influencing factors to examine the effects of GIDs on firm performance.

We selected the effective tax rate (*ETR*) to measure tax burden. This variable not only measures the extent of the tax incentives received by firms but also includes pretax deductions and concessions. The tax incentive scale developed by Dyreng et al. (2010) was used to measure *ETR*.


(5) $$\begin{array}{l} ETR=\frac{TE-DTC}{PTI+DV-IG+CD+CBI} \end{array}$$

In Equation (5), the numerator *TE* − *DTC* represents the difference between income tax and deferred income tax, and the denominator represents the pretax accounting income adjusted using China's effective tax calculation method; *PTI* represents pretax income, *DV* represents the seven impaired assets disclosed in the current period, *IG* represents investment gains, and *CD* and *CBI* represent the cash dividend and bond interest received by the firm, respectively.

Financial subsidies (*Subsidy*) were calculated by dividing total government subsidies for the current period by sales using Equation (6), where *Government Subsidy* represents the total government subsidies received by the firm in the current period and *Sales* represents the operating income in the current period.


(6) $$\begin{array}{l} Subsidy=\frac{Government\;Subsidy}{Sales} \end{array}$$

The cost of equity financing is relatively high. Therefore, the financing advantage of firms can typically be determined by observing their back loans. In this study, we calculated the financing advantage (*BankDebt*) by dividing bank loans received in the current period by total assets using Equation (7), where *Bank Loan* represents the total bank loan received by the firm in the current period and *Assets* represents the total assets of the company.


(7) $$\begin{array}{l} BankDebt=\frac{Bank\;Loan}{Assets} \end{array}$$

To account for the lag effect of the loss of political resources stemming from GID resignation, we applied a one-period lag when observing the panel data of *ETR, Subsidy*, and *BankDebt*. We also controlled the effects of company characteristics, stock structure, and board structure and took into account the fixed effects of industry and time by Winsorizing the continuous variables in the model by 1% at each tail. The regression results are presented in Table 7.

**Table 7:** Regression analysis on the influence mechanisms of GID on firm performance.

	**(1)**	**(2)**	**(3)**	**(4)**	**(5)**	**(6)**
	***ROA***	***ROA***	***ROA***	***ROA***	***ROA***	***ROA***
*T*	0.000	0.014^***^	−0.004^***^	−0.004^***^	0.002	−0.003
	(0.00)	(9.70)	(−4.40)	(−2.74)	(1.56)	(−1.33)
*D*	0.002	0.002	0.002^*^	0.002^***^	0.005^***^	0.006^***^
	(1.07)	(1.43)	(1.68)	(2.70)	(2.65)	(3.82)
D*T	0.001	0.000	−0.001	−0.002	−0.005^*^	−0.004^**^
	(0.44)	(0.32)	(−0.38)	(−1.63)	(−1.94)	(−2.18)
*ETR*	0.003	0.012^***^				
	(0.90)	(3.98)				
D*ETR	0.004	0.005				
	(0.59)	(0.85)				
T*ETR	0.000	−0.006				
	(0.08)	(−1.61)				
D*T*ETR	−0.015^*^	−0.017^**^				
	(−1.77)	(−2.49)				
*Subsidy*			0.036^*^	−0.026		
			(1.72)	(−1.47)		
D*Subsidy			−0.027	−0.052^*^		
			(−0.77)	(−1.82)		
T*Subsidy			−0.002	−0.042^*^		
			(−0.05)	(−1.73)		
D*T*Subsidy			0.023	0.018		
			(0.44)	(0.44)		
*BankDebt*					−0.003	−0.001
					(−0.43)	(−0.24)
D*BankDebt					−0.013	−0.012
					(−0.90)	(−1.10)
T*BankDebt					−0.006	0.002
					(−0.80)	(0.29)
D*T*BankDebt					0.014	0.008
					(0.91)	(0.66)
*Control Variables*		Control		Control		Control
*cons	0.032^***^	−0.076^***^	0.036^***^	−0.063^***^	0.026^***^	−0.024^**^
*R^2^*	0.001	0.352	0.004	0.373	0.005	0.354
*Adj-R^2^*	0.000	0.350	0.003	0.371	0.003	0.347
*F*	2.179^***^	208.501^***^	8.282^***^	199.566^***^	2.694^***^	47.626^***^
*N*	13,200	13,200	10,722	10,722	3,020	3,020

*R^2^ indicates Goodness of Fit, Adj-R^2^ means adjusted R^2^, F represents an F-test value, N represents the number of regression samples*.

***, **, and * *denote coefficients significant at the 1, 5, and 10% levels, respectively (two-tailed test)*.

In Column (1) of Table 7, D^*^T^*^ETR achieved a significant and negative correlation (10% level) with *ROA* (−0.015). After adding the control variables in Column (2), D^*^T^*^ETR retained a significant and negative correlation (5%) with *ROA* (−0.017). These results suggest that after GID resignation, the loss of tax incentives (increase in *ETR*) negatively affected firm performance. After controlling all other factors, a 1% increase in *ETR* reduced by firm performance by roughly 1.7% after GID resignation. In Column (3), D^*^T^*^Subsidy achieved a positive correlation with *ROA*, yet the correlation failed to achieve significance (0.023). After adding the control variables in Column (4), D^*^T^*^Subsidy retained a positive correlation with *ROA*, yet the correlation remained non-significant (0.018). Similarly, in Column (5), D^*^T^*^BankDebt achieved a positive correlation with *ROA*, yet the correlation failed to achieve significance (0.014). After adding the control variables in Column (6), D^*^T^*^BankDebt retained a positive correlation with *ROA*, yet the correlation remained non-significant (0.008). These results suggest that the decrease in financial subsidies and bank loans after GID resignation may negatively affect firm performance. However, these influences are not obvious. Although the empirical results indicate otherwise, we continue to speculate that the loss of tax incentives stemming from GID resignation was not the only influence mechanism of GID on firm performance. We intend to test this speculation in a future study. The regression results for *TobinQ* were similar to those for *ROA*.

## Robustness Testing

### Placebo Testing Method

Because of the long lag between GID resignation and firm performance, it is likely that the change in firm performance in the treatment and control groups is not really caused by the promulgation of the *Opinions*. To address the exclusivity of the treatment variables on the output variables, we adopted a placebo testing approach (Eissa and Liebman, 1995; Bergman and Nicolaievsky, 2007) by selecting years that were completely unaffected before the promulgation of the *Opinions*. First, we fabricated a treatment group and a control group, and assumed that the *opinions* were issued on January 1, 2010, so that the dummy treatment period is 2010–2012 and the dummy control period is 2007–2009. The panel data with a total of 7,910 sample observations for the 6 years from 2007 to 2012 are selected to re-run the model (4). The regression results are shown in Table 8.

**Table 8:** Placebo testing: regression analysis on GID resignation and firm performance.

	**(1)**	**(2)**	**(3)**	**(4)**
	***ROA***	***ROA***	***TobinQ***	***TobinQ***
*T*	0.011^***^	0.011^***^	0.230^***^	0.391^***^
	(12.88)	(7.24)	(6.99)	(7.10)
*D*	0.001	−0.000	−0.001	0.099^***^
	(1.21)	(−0.09)	(−0.02)	(3.21)
D*T	0.002^*^	0.003^**^	0.108^**^	0.097^**^
	(1.68)	(2.38)	(1.98)	(2.35)
*Lnsize*		0.008^***^		−0.604^***^
		(29.21)		(−64.77)
*Lev*		−0.079^***^		−1.489^***^
		(−56.52)		(−30.00)
*Growth*		0.000		0.000
		(1.33)		(0.74)
*Dual*		0.002^**^		0.071^***^
		(2.16)		(2.62)
*Soe*		−0.007^***^		−0.098^***^
		(−10.48)		(−4.45)
*Lnbrdsize*		0.003^*^		0.195^***^
		(1.75)		(3.34)
*Indratio*		−0.028^***^		1.921^***^
		(−4.77)		(9.32)
*Indplace*		−0.003^***^		−0.102^***^
		(−5.37)		(−4.98)
*Ind*		Control		Control
*Year*		Control		Control
_cons	0.026^***^	−0.088^***^	1.937^***^	13.881^***^
*R^2^*	0.017	0.306	0.006	0.430
*Adj-R^2^*	0.017	0.304	0.006	0.429
*F*	107.325^***^	184.869^***^	38.266^***^	317.231^***^
*N*	7,910	7,910	7,910	7,910

*R^2^ indicates Goodness of Fit, Adj-R^2^ means adjusted R^2^, F represents an F-test value, N represents the number of regression samples*.

***, **, and * *denote coefficients significant at the 1, 5, and 10% levels, respectively (two-tailed test)*.

The regression results concerning the effects of GID resignation on firm performance after the promulgation of the “virtual” *Opinions* tested using the placebo testing approach are presented in Table 8. The same DID model was adopted in this test. We manually shifted the promulgation of the *Opinions* to January 1, 2010 and selected a sample interval that was completely unaffected by the policy. All treatment effect coefficients (D^*^T) were positive, which was the complete opposite of the first regression analysis. These results indicate that the negative effects of GID resignation on firm performance were caused by the promulgation of the *Opinions*, validating the robustness and reliability of the research findings.

### Changing the Measuring Method of Firm Performance

In the earlier analysis, we used ROA and Tobin's *Q*-value to measure firm performance. However, because the robustness of research findings typically relies on the reasonable and reliable section of research variables, we performed the following steps to test robustness. (1) We referenced the methods adopted by Adams et al. (2005) and Cheng (2008) to measure firm performance, selecting return on equity (ROE) and return on sales (ROS) to measure firm performance. (2) The sample firms were distributed across 21 industries; to minimize the effects of industry differences on our research findings, we adopted the industry-median adjusted return on assets (IROA) and Tobin's *Q*-value (ITobinQ) as the proxy variables of firm performance. (3) With reference to Fan et al. (2007) and Cohen and Zarowin (2010), we selected the change rate of total return on assets (ΔROA) and the change rate of total return on sales (ΔROS) as the main explained variables for firm performance. These three measurement methods of firm performance were incorporated into the regression model, and no significant differences were observed.

### Using a Fixed Effect Model to Perform a Duplicate Regression Analysis on the Panel Data

Quarterly panel data were used for a pooled regression analysis. In robustness testing, we converted the pooled data into balanced data and controlled the fixed effects to eliminate the individual heterogeneity differences of the firms. Subsequently, we revalidated the model using the 14 sets of balanced panel data before and after the promulgation of the *Opinions*. The results were consistent with the previous results, with no substantial differences observed.

### Adjusting the Error of DID Estimates

We adopted the DID method to estimate the model. Bertrand et al. (2004) asserted that DID models produce low standard deviation values for the equation estimations, which may be associated with the sequence autocorrelation of the data, leading to over-rejection of the original hypothesis and overestimating the significance of the DID estimates. We identified the following methods to adjust the error of DID estimates: (1) Distinguishing the time-series data into two periods before and after the policy stimulus, ensuring that all sample firms have the same probability of treatment. This method was obviously unsuitable for the panel data used in this study. Therefore, it was discarded. (2) Applying randomization inference during regression analysis. This method not only corrected the errors in the DID estimates but also eliminated the variance caused by the sample size and ensured randomness. Therefore, we adopted randomization inference to validate the DID model. First, we sorted the 2,249 publicly trading companies in ascending order based on their codes. Then, the companies were sorted into groups of 300 in increments of 30 (i.e., the first group contained companies 1–300 and the second group contained companies 31–330). A total of 66 groups were formed and independently incorporated into Model (4) for regression analysis. The mean estimation value of each analysis was extracted and tested to determine whether it was 0. Compared with the results in Table 7, the regression coefficients and significance levels of the main variables exhibited no significant differences, validating the robustness of the research findings.

## Conclusion and Implications

GIDs were forced to resign after the promulgation of the *Opinions* by the Organization Department of the CPC. We used this exogenous event in a transition economy to conduct a natural experiment in which a DID model was developed to estimate the effects of GID resignation on firm performance. The findings indicated that the firms where GID resignations occurred experienced a steeper drop in firm performance than those without GID resignations, and that strong external governance environments mitigated the negative impact of GID resignation on firm performance. We further investigated the mechanisms by which GID resignation influenced firm performance, finding that after the resignation of GIDs, the loss of company tax incentives directly and negatively affected performance. This was the primary influence pathway by which political resources on which firms rely heavily for development were lost after GID resignation. In addition, GID resignation also reduced financial subsidy income and bank loan amounts. However, the effects of these factors on firm performance were less obvious. The findings of this study indicate that GIDs largely served as resource providers in publicly trading companies rather than in the supervisory or advisory role of general IDs.

Despite its prompting a wave of GID resignations, the effectiveness of the *Opinions* remains unclear. In this paper, we elucidated the effects of GIDs on firm performance and corporate governance and examined the implementation effects of the *Opinions*, providing evidence to support the Organization Department of the CPC's decision to ban GIDs, and validating the practical significance of the efforts of the Central Committee of the CPC in enforcing strict governance. The promulgation of the *Opinions* enabled us to test our hypotheses using a natural experiment. We believe that GIDs facilitate the governance of publicly trading companies by providing firms with political resources, serving more as “government officials” in firms rather than as IDs.

The promulgation of the *Opinions* of the Organization Department of CPC triggered a wave of resignation of GIDs of listed companies in China, but the implementation effect and strength of the *Opinions* are still unclear. In this paper, we clarify to a certain extent the impact of officials serving as independent directors on company performance and corporate governance, and examine the real implementation effect of the *Opinions*. At the same time, the promulgation of the *Opinions* has created good natural experimental conditions for the empirical study of this paper. In this study, we believe that GIDs in the governance of listed companies can be the “icing on the cake,” and the value of its existence is to bring political resources to the company, and more play the role of “official” rather than “independent director.” The findings of this paper reveal the phenomenon of “Political-Business Spin” in China, which has some implications for developing countries, represented by China, to improve the independence of the board of directors and the corporate governance.

## Data Availability Statement

The original contributions presented in the study are included in the article/supplementary material, further inquiries can be directed to the corresponding author.

## Author Contributions

TZ was the main creator of the paper, responsible for writing the paper and data processing. YL gave great help and support to the idea of this paper. DH supported in literature review and data processing. All authors contributed to the article and approved the submitted version.

## Funding

This work was supported by Humanities and Social Science Foundation of Ministry of Education of China (Grant No. 21YJC630167), supported by the Major Program of the National Social Science Foundation of China (Grant No. 18ZDA95).

## Conflict of Interest

The authors declare that the research was conducted in the absence of any commercial or financial relationships that could be construed as a potential conflict of interest.

## Publisher's Note

All claims expressed in this article are solely those of the authors and do not necessarily represent those of their affiliated organizations, or those of the publisher, the editors and the reviewers. Any product that may be evaluated in this article, or claim that may be made by its manufacturer, is not guaranteed or endorsed by the publisher.

## Footnotes

^1^ID resignation announcements were acquired from the Wind Database. We performed a manual search and consolidated the search outcomes. For details on the data collection process, please refer to the “Research Design” and “Sample Selection and Data Source” sections in Chapter 3.

^2^We reviewed all 1,352 announcements to extract the names of the IDs and their reasons for resignation.
